# The SBCCV/SBCEC Standards and Guidelines for Perfusion Practice: A Landmark for Cardiopulmonary Bypass in Brazil

**DOI:** 10.21470/1678-9741-2019-0090

**Published:** 2019

**Authors:** Gabriel Romero Liguori

**Affiliations:** 1 Laboratório de Cirurgia Cardiovascular e Fisiopatologia da Circulação (LIM-11), Instituto do Coração (InCor), Hospital das Clínicas HCFMUSP, Faculdade de Medicina, Universidade de São Paulo, São Paulo, SP, Brazil.

Since Gibbon successfully performed, in 1953, the first cardiac surgery under cardiopulmonary bypass (CPB), the technology became one of the most important pillars of cardiac surgery. In Brazil, the history of CPB begins with Dr. Hugo João Felipozzi. Felipozzi led the research that culminated in the construction of the first CPB machine produced in Brazil and the first open heart surgery in the country, in 1956, only three years after Gibbon's feat^[1,2]^. By the same time, Prof. Euryclides de Jesus Zerbini was leading the creation of a CPB workshop ("*Oficina Coração-Pulmão Artificial*") at the Clinics Hospital of the University of São Paulo Medical School (HCFMUSP), aiming the manufacture and maintenance of CPB machines and instruments^[3]^. This workshop, which was also the fruit of Prof. Adib Domingos Jatene labor, became the seed to what is, nowadays, the Heart Institute (InCor-HCFMUSP).

Although current CPB machines do not strongly differ from the original model proposed by Gibbon and used by the Brazilian pioneers, during the last decades several improvements were proposed in order to promote better outcomes after cardiac surgery with CPB^[4,5]^. Despite the significant advances in CPB systems, complications leading to postoperative morbidity and mortality still occur. For this reason, the establishment of standards and guidelines is just as crucial for the successful implementation of CPB as the scientific breakthroughs in the field.

The formulation and enforcement of such standards and guidelines is a fundamental responsibility of medical societies. In the current issue of the Brazilian Journal of Cardiovascular Surgery (BJCVS), the Department for Mechanical Circulatory Assistance (DECAM) of the Brazilian Society for Cardiovascular Surgery (SBCCV) and the Brazilian Society for Extracorporeal Circulation (SBCEC) jointly propose standards and guidelines for the perfusion practice in Brazil^[6]^ The initiative is an international collaborative effort to propose the phased adoption of the American Society of Extracorporeal Technology's (AmSECT) recommendations in our country and serve as a compass for programs striving to improve perfusion processes^[7]^. The document focuses on the empowerment of perfusion as a profession with a focus on professional qualification and education standards, the standardization of perfusion practices, the establishment of mandatory safety devices, and the importance of non-technical skills and patient-centered teamwork.

The SBCCV/SBCEC Standards and Guidelines for Perfusion Practice are divided into two parts. The first, "Minimum Standards For Perfusion Practice in Brazil", lists seven standards identified as the minimum recommendation for perfusion practice. This section was developed as an outgrowth of an ongoing collaboration with the International Quality Improvement Collaborative for Congenital Heart Surgery (IQIC). The second, "Comprehensive Standards and Guidelines for Perfusion Practice in Brazil", was strongly based on the *Report from AmSECT's, International Consortium for Evidence-Based Perfusion American Society of ExtraCorporeal Technology Standards and Guidelines for Perfusion Practice* ^[8]^ and modified according to the Brazilian regulatory agencies' policies and recommendations. The first part of the document is a ready-to-implement set of practices which should be immediately adopted by all centers performing cardiac surgery. The second is a collection of in-depth practices to be adopted in the coming years by these same centers.

One of the most important efforts of Dr. Luiz Fernando Caneo and his colleagues while writing the document was to translate it to Portuguese. The translated version of the SBCCV/SBCEC Standards and Guidelines for Perfusion Practice will allow hundreds to thousands of perfusion professionals, which are not always familiarized with the English language, to read, understand, and apply the proposed recommendations. This effort will certainly impact the way these professionals perform their work and holds the potential to influence the culture and set new paradigms in the field. Hence, the document, although published in the English language, is accompanied by an annex containing the Portuguese version.

The Brazilian Society for Cardiovascular Surgery and the Brazilian Society for Extracorporeal Circulation endorse this document as their official position in regard to perfusion practices and strongly recommend its adoption at the earliest by all the centers performing cardiac surgery in Brazil.
